# Modeling Reconsolidation in Kernel Associative Memory

**DOI:** 10.1371/journal.pone.0068189

**Published:** 2013-08-02

**Authors:** Dimitri Nowicki, Patrick Verga, Hava Siegelmann

**Affiliations:** 1 The Biologically Inspired Neural and Dynamic Systems (BINDS) Lab, Department of Computer Science, University of Massachusetts Amherst, Amherst, Massachusetts, United States of America; 2 Institute of Mathematical Machines & Systems Problems of NASU, Center for Cybernetics, Kiev, Ukraine; 3 Program of Neuroscience and Behavior, University of Massachusetts Amherst, Amherst, Massachusetts, United States of America; McGill University, Canada

## Abstract

Memory reconsolidation is a central process enabling adaptive memory and the perception of a constantly changing reality. It causes memories to be strengthened, weakened or changed following their recall. A computational model of memory reconsolidation is presented. Unlike Hopfield-type memory models, our model introduces an unbounded number of attractors that are updatable and can process real-valued, large, realistic stimuli. Our model replicates three characteristic effects of the reconsolidation process on human memory: increased association, extinction of fear memories, and the ability to track and follow gradually changing objects. In addition to this behavioral validation, a continuous time version of the reconsolidation model is introduced. This version extends average rate dynamic models of brain circuits exhibiting persistent activity to include adaptivity and an unbounded number of attractors.

## Introduction

Memory reconsolidation (ReC) is a recently proposed process explaining the update of long-term memories in the brain. Upon activation, the memory trace enters a state of lability rendering it subject to alteration and permitting integration of new information before being restabalized, or reconsolidated. “Reconsolidation” coined by Sara in 2000 [1] has become a widely studied topic in neuroscience. Recent animal and human experiments [2]–[7] have presented overwhelming evidence supporting the existence of ReC and identified boundary conditions that characterize and limit this phenomenon [8]. ReC is postulated to strengthen, weaken or extinct memories and update them with new, relevant information. Reconsolidation draws a striking new way of understanding memory and its roles: from a computer-like reliable log, to an adaptive and active part of perception.

Recent experiments have also identified reconsolidation as a possible avenue of treatment for phobias and PTSD by effectively allowing the erasure of fear memories. These memories come about through classical conditioning mechanisms that pair aversive stimuli (unconditioned stimuli – US) with co-occurring, once neutral stimuli (conditioned stimuli – CS). This coupling is the basis for anxiety disorders and PTSD. The most common treatment for fear related disorders is exposure therapy. Exposure therapy leverages extinction learning mechanisms to create a second safety memory that competes with and suppresses the fear response [9], [10]. This technique, however, does not fully erase the fear memory, allowing it to spontaneously reappear [11]. Reconsolidation has been demonstrated as a possible method of completely erasing fear associations. In several experiments, fear memories in previously conditioned rats were reactivated, returning the memory traces to labile states. Protein synthesis inhibitors or beta-adrenergic receptor antagonists were then injected into the amygdala, blocking the reconsolidation process. This process resulted in extinction of fear and was not subject to spontaneous recovery [4], [5], [12], [13]. Cases of reconsolidation of fear memories have also been demonstrated in humans. In these experiments, subjects were exposed to stimuli, which reactivated the fear memory trace rendering it labile. Rather than pharmacological intervention, the normal reconsolidation process was disrupted with competing information which resulted in the memory being updated [14], [15].

We propose an adaptive memory model that is consistent with recent findings in ReC. The framework introduces efficient ways to add, remove, and update attractors. Additionally, memories can be strengthened, weakened, or extinguished by controlling the attractor radius.

Our memory model builds on an earlier Kernel Associative Memory (KAM) model [16], [17] that uses a kernel structure to efficiently compute attractor dynamics. The KAM model is an extension of the attractor based Hopfield network. It has been shown that attractor mechanisms are employed by the brain, notably in the CA3 region of the hippocampus [18]. The KAM has several advantages over previous Hopfield models including the number of attractors unbounded and independent of the input dimension, dynamic rewiring of neurons, and the ability to accommodate large real-valued inputs and attractors. This paper derives a ReC algorithm that allows KAM to hold an unbounded number of now flexible attractors, which we call ReKAM. Our approach to the modeling of reconsolidation is based on the principle of robust global update, analogous to psychological findings such as the gang effect where the update of one attractor affects neighboring attractors [19]. We also introduce an approximate ReC algorithm which changes the global updates to local ones, gaining time efficiency at the cost of precision.

The relevance of our ReKAM model is demonstrated by replicating three recently found characteristics of ReC seen in human behavioral experiments. First, ReKAM imitates a recent list-learning experiment in which human participants merged new objects into a previously learned list during retrieval. ReKAM also demonstrates fear extinction via the controllable attractor radius. The third experiment follows gradually changing objects resulting in an evolved representation. Finally, a continuous time version of ReKAM is introduced which relates the model to neurobiological studies. This version extends the capabilities of the continuous-time Hopfield network [20] commonly used to model average firing rate dynamics [21], [22] of adaptive persistent activity.

### Previous Reconsolidation Models

Reconsolidation's significance in explaining the dynamic properties of healthy memory has led to several mathematical models proposing to explain the process. The first ReC model [23] extended the Hopfield model to allow attractors to evolve through weight decay and Hamming-distance terms. Our ReKAM also allows attractors to evolve, but since our attractors lie in high dimensional space, the number of memories is unbounded and inputs are realistic, thus modeling reconsolidation in a more relevant and technologically practical way.

The second ReC model to be introduced, called Reconsolidation Attractor Network (RAN) [24], takes the approach that attractors do not have to lie in input space and hence an unbounded number of memories are possible. The architecture of the RAN is layered. Attractors appear in the upper level separate from the neural flow and input space. Our ReKAM builds on the same concept of attractors not lying in input space, but it also draws from Hopfield-like networks for mathematical completeness of attractor dynamics.

The third model presented in [25] is designed to reproduce extinction of fear memories. Like the first model, it is also based on the classical Hopfield network. Attractors can be extinct when an additional binary variable which represents the anisomycin (consolidation-inhibiting) drug is set to 0. Our ReKAM is the only memory model demonstrating all known ReC properties as opposed to a particular architecture demonstrating only one facet of the ReC process; it is also the only one that describes reconsolidation of large memories with real world stimuli.

### Modeling with Kernels

Our ReKAM model is based on our KAM architecture [17]. Kernel representations were introduced by Vladimir Vapnik to the field of Machine Learning when he showed how to transfer input data to a high-dimensional data space called 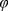-space (phi-space). The data is classified in 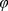-space and then projected back to the original space resulting in the most efficient, optimal, non-linear separation. This is achieved by using the kernel property: a scalar kernel function applied to two inputs is equal to their product in the 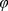-space: 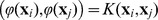. This kernel property is the basis of Support Vector Machines (SVM), regarded as the most efficient supervised classifiers [26].

Support Vector Clustering (SVC) was introduced in a joint work by the third author's research group and Vapnik. SVC is an unsupervised extension of SVM (for the case when labels are not available) that groups data into clusters through kernel functions that mimic high-dimensional organization and projections [27].

In Kernel Associative Memory, we follow similar mathematics. However, here the 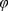-space is not abstract. Instead, it is based on the output of multiple neurons. Mathematically, Mercer kernels are no longer sufficient. We define the strong Mercer kernels that provide the condition needed to load an unbounded number of attractors (See Materials and Methods 4.4). The use of both low-level and high-level spaces is an effective mathematical way to describe both the synaptic changes of neurobiological memories as well as the behavioral effects of cognitive memories.

### Model for Reconsolidation based on KAM

The practical advantages of our ReKAM model include an input space that can be composed of continuous valued vectors rather than binary ones, a number of attractors that is independent of the input dimension, and a variable input length where longer and shorter input vectors are learned with no a priori bound. Furthermore, attractors are efficiently loaded, deleted, and updated.

We briefly describe the KAM which is the basis of our ReKAM model (a complete description is given in [17]. Let 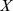 and 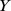 be matrices whose columns represent the input and output space of the memories. Memories are defined by the transformation on these columns through the projective operator. We transfer the input to the higher 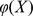 space (as explained in previous sectionf), so that the current transformation is now : 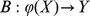. A connection matrix 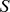 is defined as:
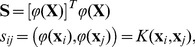
(1)


Memory loading is defined by
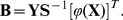
(2)and recall of input 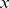 by the iterations:
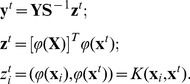
(3)where the first iteration is initialized with 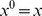, each iteration ends with applying any sigmoid-like activation (bounded monotonically increasing) function coordinate-wise to 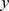: 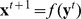, and the iterations stop when update is under a chosen threshold. The KAM can be depicted as a neural network, as explained in [17].

## Results

### Model for Reconsolidation and Extinction: ReKAM

Unlike the traditional Hopfield networks, where attractors lie in input space, our ReKAM's attractors (stemming from the KAM architecture, see last subsection in Previous Work) lie in a high dimensional manifold. While a Hebbian networks' (e.g., [23]) synaptic matrices compose a linear space, our use of the efficient pseudo inverse learning method gives rise to Riemannian manifolds in the attractor space. An unbounded number of attractors can exist in the higher dimensional space. Between every two points in a Riemannian manifold there exists at least one geodesic that has a minimal length of all curves joining the two points. The geodesic is analogous to the shortest straight line between two points but in a nonlinear space. Updating an attractor toward a new input is calculated along a geodesic between the new input and the given attractor it recalled. Our ReC algorithm with this manifold makes the memory update global, and capable of representing psychological properties such as the gang effect. This global update is more expensive, although more accurate, and we provide another local algorithm which is faster and just a bit less general. Comparisons between the architectures are provided both for time analysis (in this section below) and in the result “Updating Memories Incrementally”.

#### The global geodesic ReC Algorithm

We propose a memory update algorithm that assumes that every ReC update has a global effect. Mathematically it is based on geodesic computation in the Reimannian manifold representing the memory attractors. The metric structure of this manifold and a comparison with the special case of the Grassmann manifold are derived in Materials and Methods (4.1–4.3).

Suppose that we have an initial memory 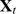 that contains 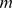 patterns (concepts) 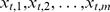. We then obtain 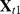 by replacing one of the attractor patterns 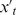 with a new stimulus 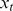 that recalls it. The distance between **X
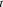
** and **X
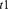
** can be interpreted as a measure of the amount of “surprise” that the memory experiences when it meets a new stimuli. To track these changes, we build a geodesic 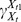 joining **X
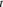
** and **X
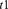
** on the manifold and take a new point 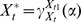. Here 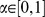 is a step parameter related to the size of a shift during each update. When 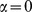, the memory remains at **X
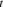
**, when 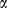 = 1, the memory is changed to **X
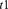
**.

**Figure 1:**
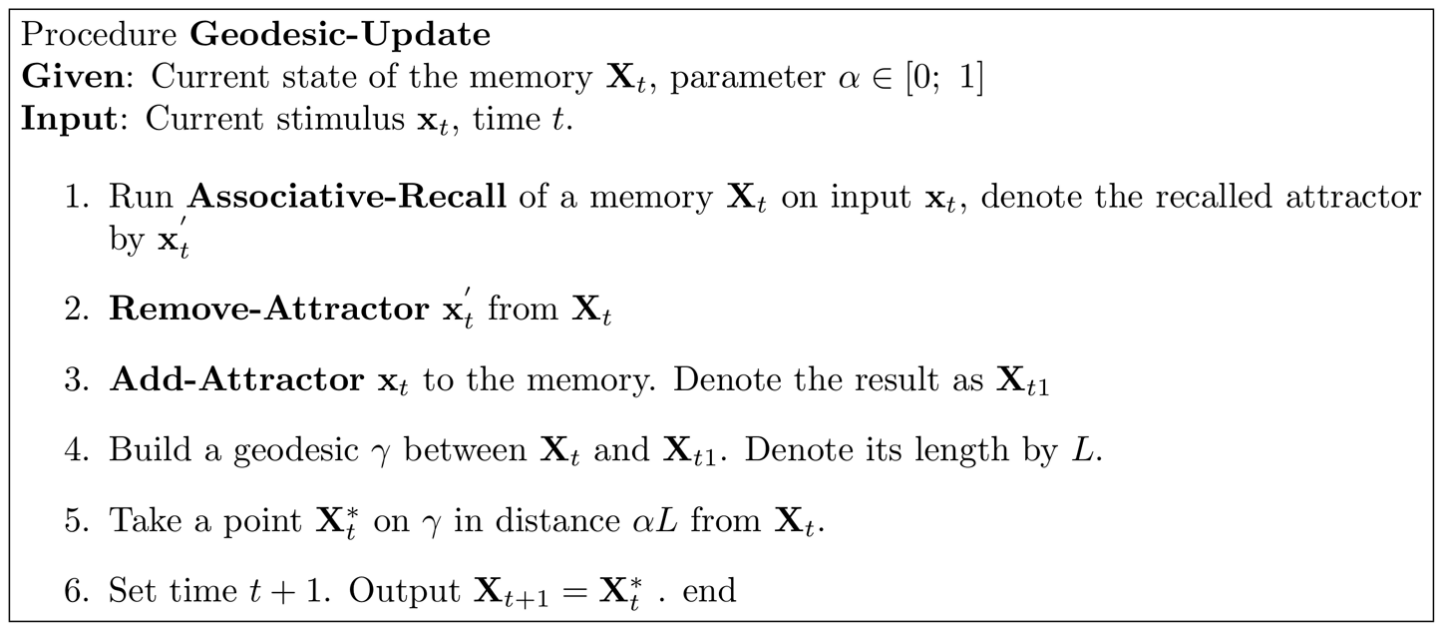
Algorithm of Geodesic Update.

Using the same process, when a stimulus 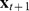 appears we can track the change from **X
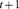
** to 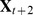. The process the continues for future stimuli. The algorithm of memory update using geodesics is shown in Fig. 1. The exact geodesic calculation is described in the Materials and Methods. Its complexity depends on the optimization algorithm used. The dimension of our manifold is 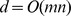. A “typical” gradient calculation would require 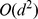 operations. The gradient-like minimum search calculation has complexity 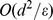 where 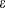 is the required tolerance [28]. This leads to complexity 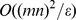 [29]. However, with derivation-free optimization techniques which do not require explicit gradient calculations, we can reduce this complexity estimation to 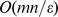.

#### The Local Approximate ReC Algorithm

The exact computation of geodesics may be resource consuming especially for high dimensional data. Here we develop a simplified ReC algorithm with local rather than global updates to attractors. In this linear approximation, we simply replace the geodesic with a straight line in the coordinate space.

**Figure 2:**
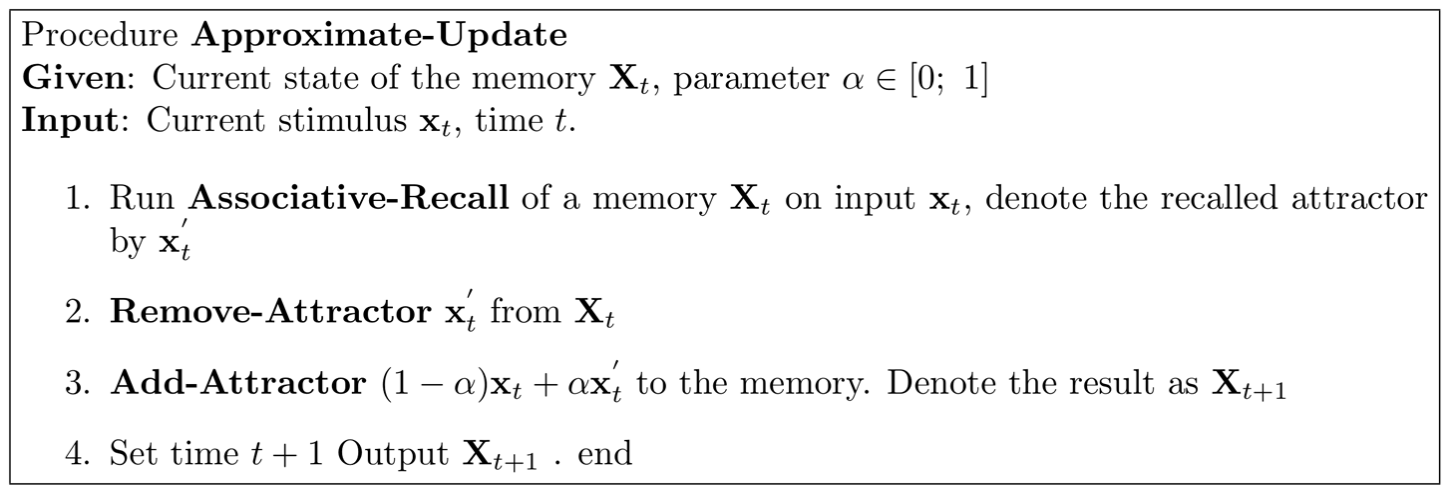
Algorithm of Approximate Update.

This leads to the Approximate-Update algorithm in Fig. 2. The approximation algorithm's complexity is 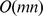, equivalent to the derivative-free version of the geodesic algorithm. However, the approximation algorithm is much easier and faster to implement. Because it requires only a few operations per element, the complexity does not depend on the tolerance.

While the approximate algorithm of Fig. 2 shows only one update per reconsolidation, we can easily construct a version of this algorithm that updates any desired number of attractors. For this, we repeat step 3 for the 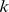 most relevant attractors with the largest values of 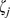 where 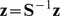, and use the value 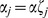 for the 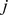-th attractor. This version of the algorithm demonstrates gang effect properties by updating neighboring attractors.

The approximation error is bounded by the following theorem:

**Theorem 1** *Let* 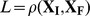. Denote 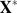 *as the solution given by the geodesic algorithm and 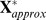 as the solution given by the approximate algorithm. There then exists a constant 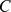 such that*

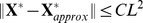



**Proof:** Let 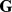 be a metric tensor on 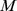 dependent on coordinates and 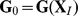. The straight line 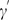 between 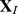 and 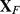 is a geodesic in the flat space with a constant metric form 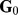. Since 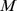 is a twice differentiable manifold, 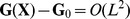 along the geodesic 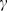 that lies between 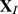 and 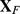. Denote 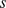 as the distance between the starting point 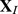 and a given point 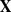 along the (geodesic) curve. 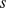 is called the arc length (see remark 1 below, [30], or other textbook on Riemannian and differential geometry). Because 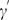 is a secant of the 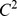 curve 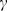 in the coordinate space, when 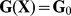, there exists a constant 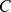 such that for the given arc length *s*, 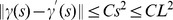


**Remark 1** *Arc length could also be defined as a parameterization of a curve 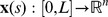 such that* 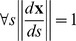.

#### Controllable Attraction Radius

As part of the ReKAM architecture we include a mechanism for altering the size of an attractor's basin of attraction. This affects the probability of recalling an attractor. As the attraction radius goes to zero, the attractor will never be recalled. This is analogous to extinction.

**Definition 1** *A Kernel* 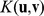 *is called* ***uniform*** *if it depends only on the difference* 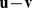


If the kernel network has a uniform kernel, Then the attraction radius can be controlled. Assign the *scaling factor* 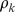 to the 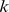-th attractor. We can then divide the 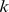-th entry of 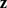 by 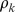 where 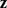 is the temporary vector used in the recall algorithm of ReKAM.
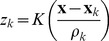
(4)


This causes the attraction basin to be scaled by 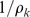.

### Model Verification with Human Experiments

We verified our model's ability to describe reconsolidation by comparing the dynamics of our model to those observed in humans. The first experiment simulates the effect of reconsolidation on episodic memories. The second demonstrates the model's capability to replicate extinction. The third follows memory changes created by the gradual altering of the associated input.

#### List Learning

**Figure 3:**
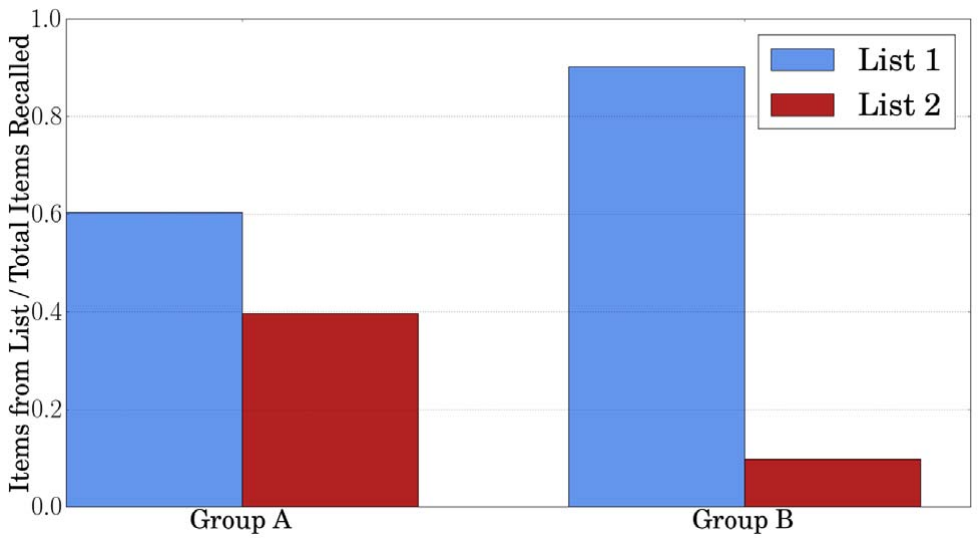
Results of the original list learning human experiment [31]. Group A received a reminder cue before learning List 2. This resulted in the List 1 memory becoming labile and updated by integrating some of the new items from List 2. Group B did not receive this reminder and these intrusions were not seen. For our analysis, results were normalized for each group by dividing the number of items recalled per list by the total number of items recalled in both lists together.

We first replicate a human experiment investigating reconsolidation of episodic memories [31]. In the original experiment, participants were split into two groups (A and B). On Day 1, both groups learned a list of 20 objects (List 1) that were associated with a blue basket. On Day 2, Both groups learned a second list of 20 items (List 2). Before learning, group A received a reminder of List 1 in the form of the blue basket; group B did not receive any reminder. On Day 3 both groups were tested on their ability to retrieve List 1. Group A made more errors confusing List 2 items into List 1 than Group B did (Fig. 3). When the experiment was repeated to test recall of List 2, both groups performed equally well.

In our simulation, all objects were shown as images, rescaled to 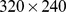 pixels. Note that the ability of the model to handle large colored images is already beyond the standard Hopfield model used in previous work. Images were represented as real-valued vectors with components 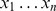. We added an indicator variable 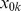 to each item, 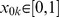 where 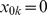 denotes that the object is unrelated to the 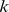-th list, and 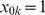 means that the object belongs to this list with 100% certainty.

For computational efficiency, we took the variant of the Gaussian kernel:
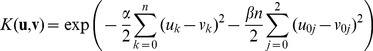
(5)where 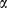 and 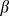 are tuned to balance between the data vector and the list indicator components.

In our simulation we modeled the two groups. For each group we created 40 initial attractors corresponding to the items in both lists. In group A we gradually shifted the value of 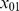 of each item towards 1 when this item was recalled with the blue basket reminder in the background to simulate the effects of reconsolidation. In group B, these updates were not performed. For both groups we tested the memory in recall mode inputting 1000 new vectors per list by taking the attractor and adding uncorrelated white noise (intensity equaled 10% of data STD). For all query vectors, we set the 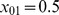 as the initial value.

Using our model, we found an exact correspondence between our simulation and the human experiment for values of 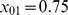 for Group A and 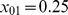 for Group B (see Table 1).

**Table 1:** Comparing the results of actual human experiment to our model's simulation.

Human Results		
Group	A	B
List 1 %	60.399	90.180
List 2 %	39.600	9.819

Simulation Results		
*x*_01_	0.75	0.25
List 1 %	60.287	90.105
List 2 %	39.713	9.895

By normalizing the data by the total number of items recalled over both lists, our simulation matched exactly with Group A and Group B of the human experiment.

**Figure 4:**
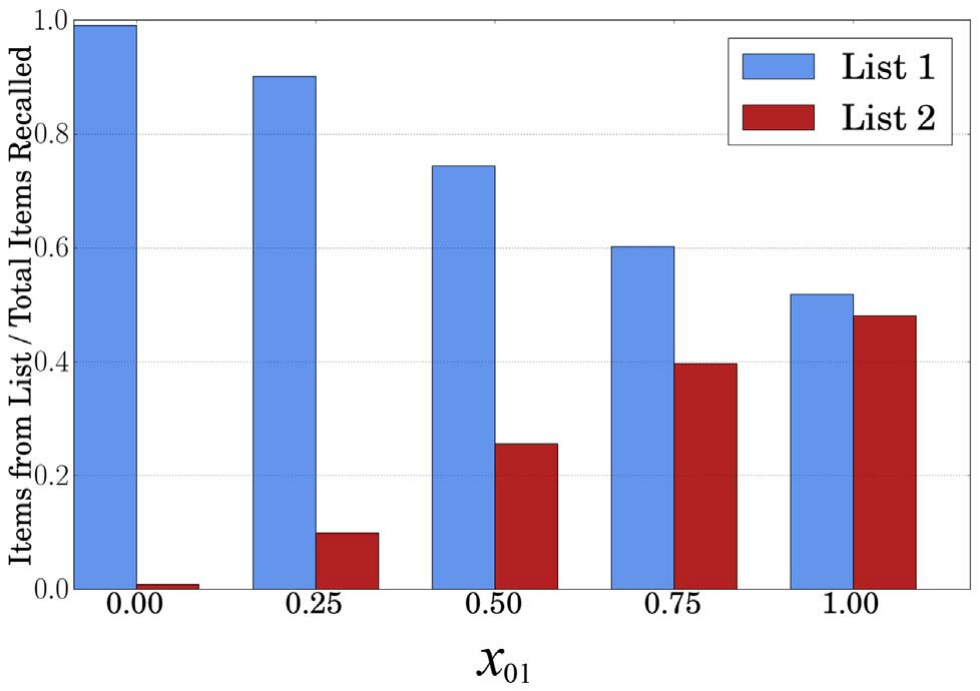
Results of our models simulation of reconsolidation on the list learning experiment for different values of the reconsolidated parameter 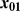. **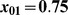 and 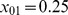 exactly matched the results of Group A and Group B of the human experiment.**

We next simulated more values of 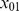 which could arise for varying levels of reconsolidation due to differing experimental procedures, memory type, etc. (Fig. 4).

#### Extinction

Many recent experiments have demonstrated the effects of fear extinction in both humans and animals – e.g. [32], [33], [34]. Numerical simulations with Hopfield memory and Hebbian-like learning were presented in [25]. Our model has a far larger number of far more detailed memories than previously modeled.

**Figure 5:**
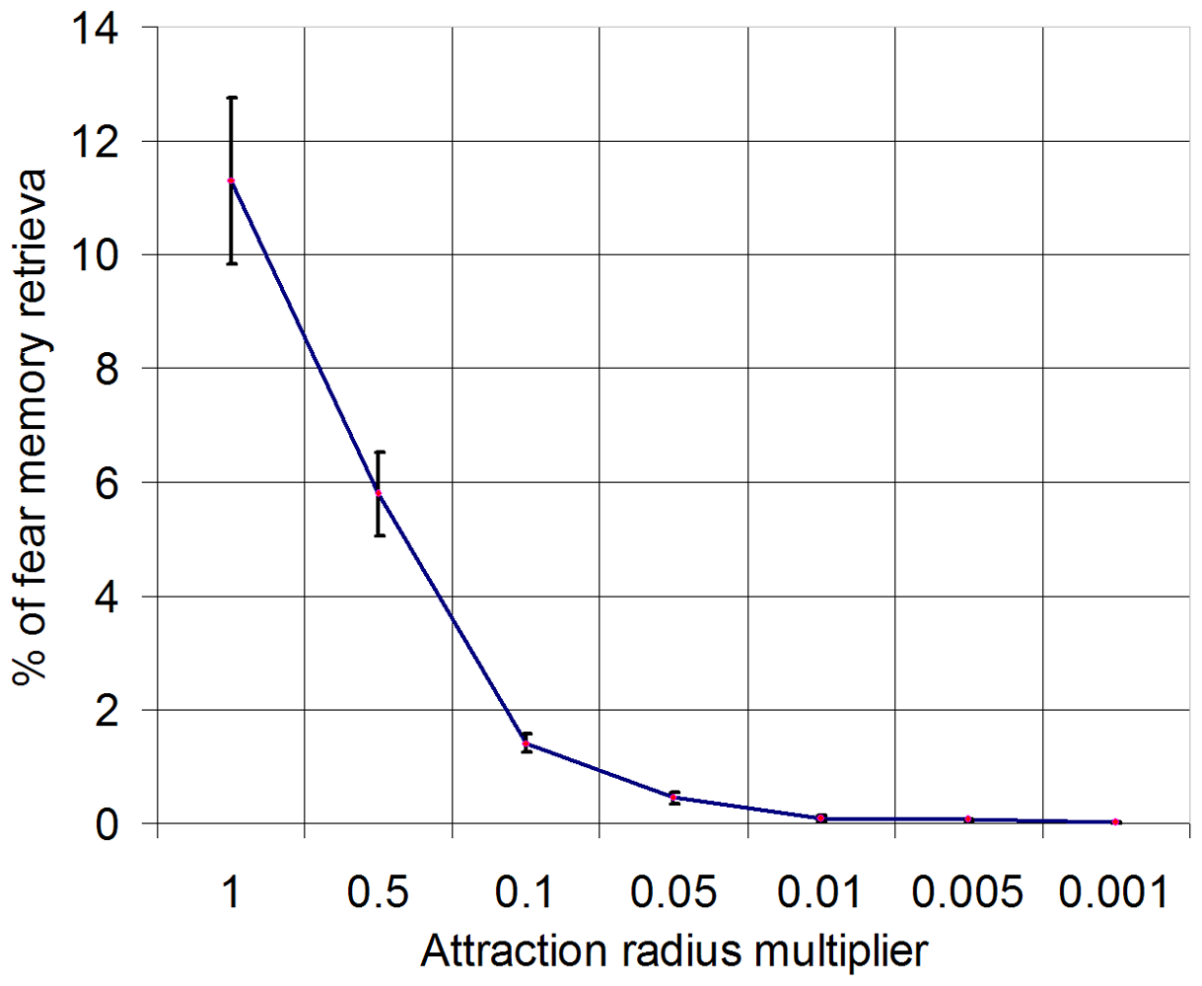
Results of extinction experiment showing the probability of shock memory retrieval as the attraction radius multiplier is varied. Each point represents the average of 1000 random input trials as well as their standard deviation. Note that the scale of the x-axis varies.

We propose to model extinction as a reduction in the attractor's radius. To demonstrate, we created a kernel network that memorized 10 images. All images were scaled to 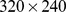 pixels. One of the images was randomly chosen to be a “fear” (shock) memory. In our procedure, the scaling factor for the “shock” attractor was gradually decreased. This process is analogous to the weakening of the memory occurring through reconsolidation during extinction training. For each scaling factor value we measured the frequency of retrieval (recall) of the shock memory on 1000 random inputs (Fig. 5). The decreasing attraction basin radius effectively extinguishes the fear memory trace as its probability of recall goes virtually to 0.

#### Updating Memories Incrementally

In an experiment testing the incremental changes of gradually morphing memories [35], participants learned to recognize four faces as “friends.” One face was morphed incrementally over a period of days. When the face morphed slowly, participants continually recognized the morphed face as their original friend. By the end of the process, the morphed face was recognized as a friend while the original face was not. The results demonstrated merging of the source and the new face. However, this effect was only observed when the faces were changed gradually, demonstrating that the order in which morphing took place was crucial. A gradual, subtle change was needed to allow for reconsolidation to occur.

In our previous work [17] we published a numerical experiment with morphing face images that replicated the previous result described above. Attractors in the KAM were gradually morphed following the slowly changing face inputs.

**Figure 6:**
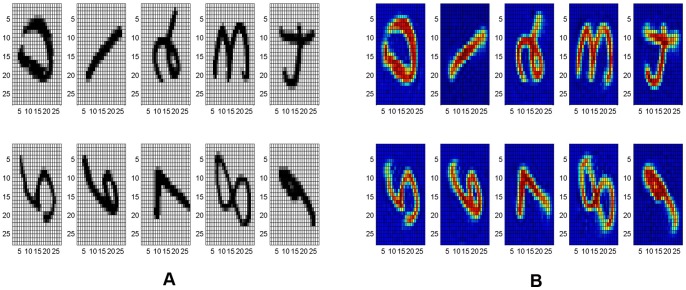
Example of rotated digit inputs (A) and corresponding attractors (B) during reconsolidation.

Here we present a similar experiment aimed at examining the network's ability to track images varying gradually over time. Additionally, we compare the performance of the exact and approximate ReC algorithms for this manipulation. We created a training set consisting of 9,000 rotated digits. The rotated digits were created from 100 original MNIST handwritten digits (10 per class from ‘0’ to ‘9’). Digits were 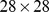 pixel grayscale images which we rotated counterclockwise from 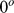 to 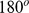 (Fig. 6).

We applied principal-component (PC) preprocessing without considering any specific handwritten digit optimized feature extraction techniques. We took the first 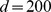 PCs which contain 96.77% of the variance. For computational efficiency, the kernel we chose was:
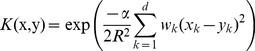
(6)where 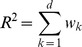, and 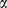 is a bias parameter. This is a Gaussian kernel dependent on a weighting metric. The weights were chosen as:
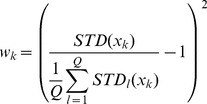
(7)We also tried:
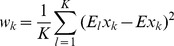
(8)Where 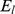 and 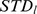 are the expectation and standard deviation over the 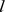-th class, and 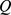 is the number of classes. Formula (7) yielded better results.

**Figure 7:**
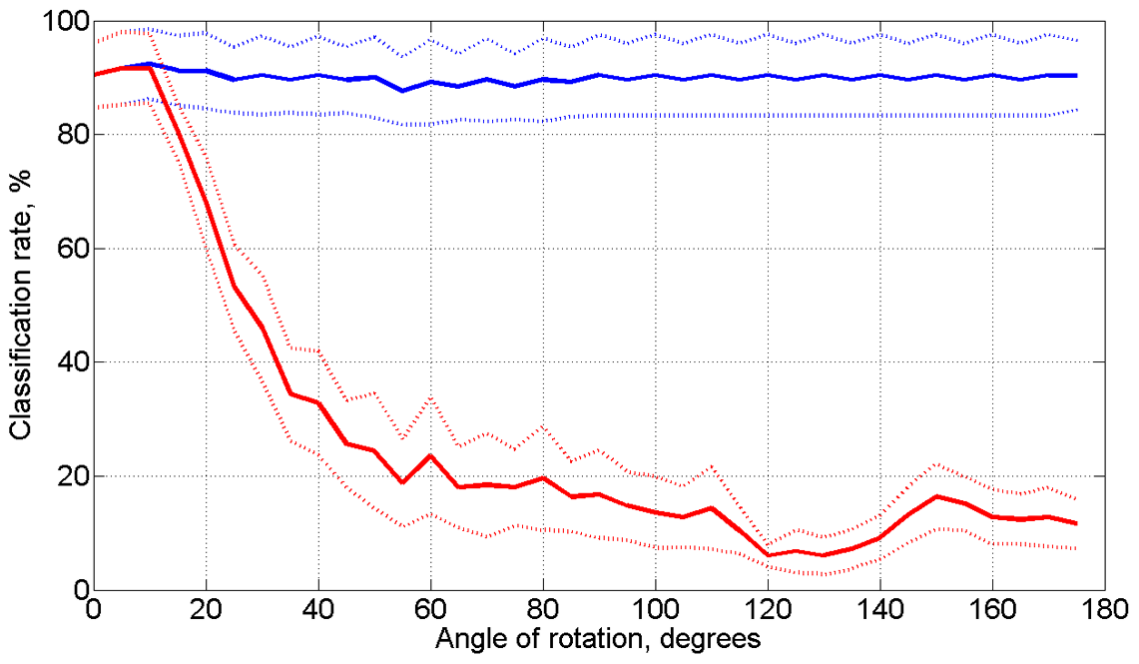
Results of the digit rotation experiment . The solid lines show the average accuracy over all classes for rotations between 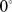 and 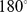. The blue line shows the accuracy while using reconsolidation. The red line shows the corresponding accuracy while not reconsolidating. The dotted lines surrounding the solid lines refer to the standard deviations.

Evolution of the classification rate over time for the digit rotation experiment is shown in Fig. 7 with confusion matrices in Fig. 7. The exact reconsolidation algorithm achieved a recognition accuracy of 96.4+/−0.43%. Results for the local approximate algorithm were 96.32+/−0.26%. The algorithm without reconsolidation performed significantly worse (see Fig. 7). The CPU time was 142 sec for the approximate algorithm and 54.7 min for the exact geodesic reconsolidation on Intel Centrino Duo 1.4 GHz CPU with no parallelism, in the Matlab environment. The average relative error in attractors was 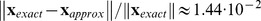.

**Figure 8:**
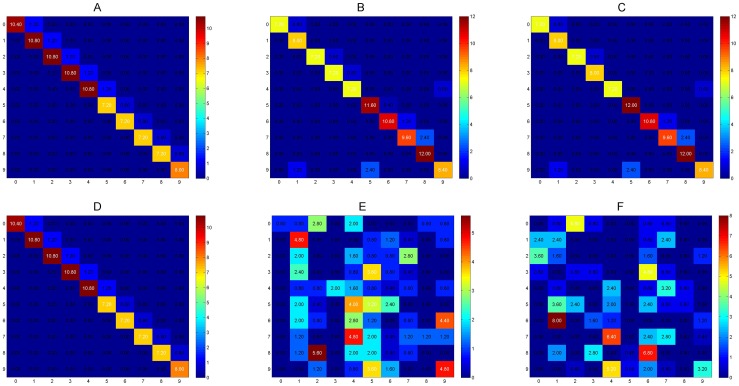
Confusion matrices for rotating digit experiment showing predicted labels vs true labels where a correct classification appears along the diagonal. A, B, and C show reconsolidation with 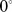, 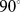, and 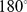 rotations. D, E, and F show results without reconsolidation for 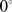, 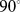, and 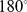 rotations. Warmer (more red) colors refer to higher number values. The reconsolidation algorithm provides stable clustering even under changing conditions (presented as rotation in this experiment). Without reconsolidation, the memories are unable to track these changes.

When inputs were shuffled randomly, gradual reconsolidation was unable to occur. We note that because we are testing on a handwritten digit dataset, there are variations between each test digit: while an ideal number 6 rotated 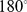 would be equal to an ideal number 9, this will very rarely be the case with the variable hand written digits. Results of no reconsolidation for digits rotated at 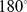 (Fig. 8–C) shows that digits such as 0, 1, and 8 remain mislabeled while, to a human eye, these would seem the same. It is possible that using a preprocessing technique specifically designed for hand written digit recognition may allow the system to generalize these specific cases to a greater degree. However, even under these conditions, the reconsolidation algorithm works effectively and allows for accurate classification under constantly changing inputs.

### Continuous-time ReKAM Models Firing-Rate Dynamics

Up to here, we described the discrete-time form of associative recall. We next relate the ReKAM to biology by introducing a continuous-time version of the kernel memory and comparing it to other firing-rate models. It is important to note that the nature of the time (discrete or continuous) is involved only in dynamical systems of recall, not in the reconsolidation phase. Any step of the reconsolidation (both exact and approximate) depends only on the input and the attractors, not on the time. For this reason both the exact and approximate algorithms of ReC work in continuous time.

The Hopfield equation for the 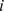-th neuron is:
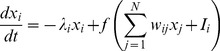
(9)where 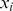 is the output of the 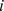-th neuron, 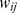 are the elements of the symmetric synaptic matrix 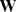, 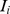 are direct external inputs, 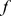 is the activation function, and 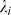 is the “relaxation rate” of the 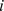-th neuron. The Hopfield equation (9) imposes linear and symmetric neuron-to-neuron interactions in the network which can be described by the synaptic matrix 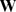. Escalating the model from discrete neurons to neural field (mean field) gives rise to the Wilson-Cowan partial integro-differential equation [36]:



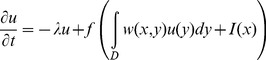
(10)If the activation function is simply the Heaviside step function, equation (10) becomes the Amari field equation [37].

If the network's activity is a Markov stochastic process (with a vector 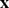), then the first-order approximation of the average firing rate dynamics is (see [38]):
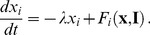
(11)


This equation can have arbitrary dynamics in contrast to (9) that has a Lyapunov function and converges to attractors.

We propose a continuous-time version of the Kernel Associative Memory that updates the recall similar to (11):
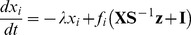
(12)where the components of 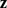 are:



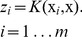
(13)The continuous ReKAM described by Equations (12) – (13) with a scalar-product kernel is isomorphic to (9) except for having the synaptic matrix W calculated by the pseudoinverse rule (not the Hebbian rule), or, equivalently, orthogonal Hopfield learning. This continuous memory inherits the Hopfield-like attractor dynamics but is more biologically relevant: the number of attractors is independent of the input dimension and rewiring of the neurons is dynamic. We propose the continuous ReKAM as a model for firing rate adaptive dynamics in the course of persistent activity in various networks in the brain.

## Discussion

While the existence of reconsolidation in human memory was once a topic of debate, the accumulation of human experimental results has led to the mechanism becoming widely accepted in the field of neuroscience. Reconsolidation has been dissociated from extinction learning, the latter of which results in a second memory trace rather than the removal of the old one [39], [40]. However, it is not yet entirely clear when or to what extent reconsolidation mechanisms will occur in a given situation. Experimental results have identified numerous boundary conditions involved in determining whether or not a memory will undergo reconsolidation [39], [40].

One such boundary condition is the amount of time between a memory's retrieval and the encountering of relevant stimuli. This time window varies depending on the animal tested [41] and in humans begins about 10 minutes after retrieval and lasts for several hours. During this time, the memory is labile and susceptible to new information or experimental interference. If the stimulus is encountered outside of this time window, reconsolidation will not occur. A second boundary condition is the age and strength of the memory trace, affecting the ease in which the memory will undergo reconsolidation. A stronger or older memory may require longer and more frequent reactivation sessions for reconsolidation to occur. A third condition, the predictability of reactivation stimulus, also plays a role in whether or not reconsolidation will occur. If a subject does not correctly predict a novel response to a stimulus, reconsolidation is more likely to occur in order to update an incorrect prediction model [42]. Another boundary condition is the “trace dominance” – when a memory stabilizes and becomes resistant to reconsolidation and certain amnesic agents.

It would be possible to extend our model in the future to include these observed boundary conditions. The addition of variables that account for time elapsed since retrieval, age of memory, and strength of memory could be implemented to allow for an accurate simulation of the boundary conditions that accompany reconsolidation. Additionally, a mechanism to account for prediction error would allow for a representation of the novelty prediction that has been shown to influence whether or not reconsolidation will occur. These additions could allow for a more accurate simulation of reconsolidation as well as a more biological learning model.

We have proposed a mathematical framework of memory reconsolidation, which demonstrates properties as seen in human studies: incremental updates, associations, and extinction. Our ReKAM memory model is far more technologically relevant than previous ones in that it is able to include real-valued inputs as well as massively long inputs; the number of memories is independent of input dimension and hence is practically unbounded. This results in a model providing both a better functional understanding of reconsolidation and the basis for a powerful technology for following changes in real world environments.

The mathematical structure has its own beauty: The kernel associative memory has an underlying structure of a Grassman-like manifold in the (feature) 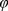-space. Since it is a curved Riemannian manifold, reconsolidation is no longer a linear update, but the creation of geodesics is required. We provided both an exact Reconsolidation algorithm as well as a more efficient one, which is local in update and does not require the exact computation of geodesics. A continuous time version of the memory is introduced with further biological relevance.

The kernel method opens the door to reconsolidation of multimodal and dynamical (temporal) memories; this is a subject of our future research.

## Materials and Methods

### Defining a Riemannian Structure for the ReC algorithm

We formulate the distance between two kernel associative networks where both networks have the same kernel and number of memories. Each network contains a different sets of memory attractors. In the 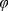-space each kernel memory is a symmetric network whose synaptic matrix is a projective operator 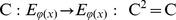. We measure the distance between two projective operators, **X** and **Y** (both of finite rank 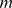), as a Frobenius norm 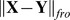. Taking into account protectiveness and self-conjugatedness of **X** and **Y**, we have:
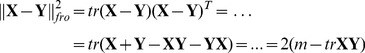
(14)


For each projective operator 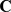, the singular value decomposition (SVD) leads to the following:
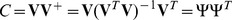



For any 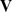 defined as above, a matrix 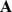 (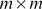) is defined as having elements 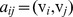, the pairwise scalar products of the memorized vectors. In matrix notation this is represented as:
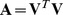
Since 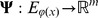 is an orthogonal operator, then



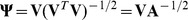



Using this template we represent 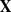 and 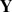 as follows: 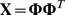, 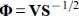; 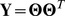, and 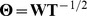. So,
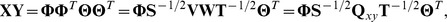
Here **Q** is an ***m
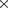
m*** matrix such that 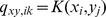. Having a singular decomposition for **XY**, we can now compute the distance as



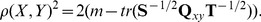
(15)The above defines a Riemannian structure for the KAM manifold.

### Pseudoinverse Memories and the Grassmann Manifold

We next relate the manifold defined by the ReKAM model to the more well-known and less complex Grassmann manifold. An associative memory with a pseudoinverse learning rule is described in [43]. This is a Hopfield-type auto-associative memory defined originally for bipolar vectors: 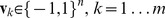. Suppose these vectors are columns of 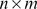 matrix 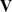. Then a synaptic matrix 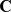 of the memory is given by:
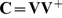
(16)where 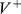 is a Moore-Penrose pseudoinverse of 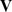. For linearly independent columns of 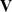, the pseudoinverse can be computed by 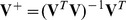 or by using the Greville formulae (see, e.g., [44]). The resulting weight matrix 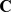 is projective, i.e. 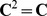 with rank 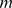.

The Grassmann manifold is a particular type of Riemannian. The Real Grassman manifold is the manifold of all 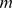-dimensional subspaces in 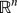 and is denoted as 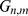. To define the Grassman manifold, we first introduce the Stifiel manifold – a set of orthogonal 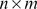
–matrices 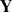, 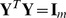 endowed with the Riemannian metric which is induced by the Euclidean norm in the space of 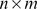-matrices. Next, we say that two matrices are equivalent if their columns span the same 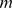-dimensional subspace. This means that two matrices 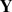 and 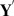 are equivalent if they are related by right multiplication of an orthogonal 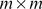 matrix 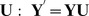. The quotient of the Stiefel manifold with respect to this equivalence relation is called *Grassmann Manifold* [45].

For each 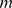-dimensional subspace in 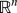 there exists a unique projective matrix 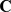 of rank 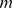, and vice versa (see [46]). Therefore a space of 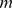-ranked projective matrices is a Grassmann manifold 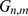. Moreover, the Frobenius norm of the difference of two projective matrices 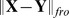 gives one possible Riemannian metric over this manifold.

The Grassman manifold emerges in our model in the special case of a scalar-product kernel. Other kernels used in our ReKAM model result in manifolds that can be considered generalizations of the Grassmann.

### Computing Geodesics for the ReC Algorithm

To implement the geodesic update algorithm, we have to efficiently compute geodesics on the kernel memory manifolds. Given the metric in explicit form (15) this can be solved as an optimization problem. Let 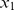 and 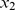 be points on manifold 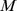 with metric 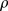. Let a point 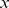 lie on the (minimizing) geodesic segment joining 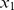 and 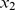. 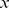 divides the segment into two parts with proportions 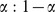. Let 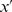 be a point which lies on the manifold but not in the geodesics. The process of finding 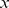 is stated as follows:
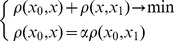
(17)


The geodesic minimizes the sum of two distances (first line). For the point 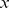 on the (minimizing) geodesic, the following inequality holds:
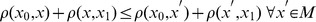
(18)


The Process (17) can be solved numerically using a Gradient Descent Method (or other first-order unconstrained optimization method). Its complexity is 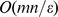 for a tolerance 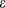. The constant here is typically large due to the hardness of gradient computation.

### Functions with Mercer Condition

The classical Kernels 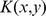 introduced to the field of Machine Learning by Vapnik [26] had the Mercer condition. That is, for all square integrable functions 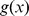 the kernel satisfied:
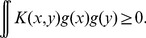
(19)


The Mercer theorem states that if 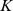 satisfies the Mercer condition there exists a Hilbert space 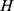 with a basis 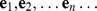 and a function 
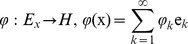
, where 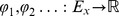, such that
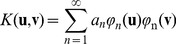
(20)and all 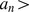
0. That is, 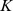 is a scalar product of 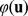 and 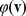


General Mercer kernels are not sufficient for creating the associative memory since our kernel memories require that all attractors are linearly independent in the feature space. Some Mercer kernels, such as the basic scalar-product kernel 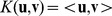, do not assure this property. The strong Mercer kernels defined for our kernel memory [17] provide linear independence of the attractors in the feature space which enables correct association.
